# Exploring factors underlying the use of nyaope in Tshwane, South Africa: A qualitative study

**DOI:** 10.4102/jcmsa.v3i1.129

**Published:** 2025-06-26

**Authors:** Doudou K. Nzaumvila, Robert Mash, Toby Helliwell

**Affiliations:** 1 Department of Family Medicine and Emergency Medicine, Faculty of Medicine and Health Sciences, Stellenbosch University, Cape Town, South Africa; 2 Department of Family Medicine, Faculty of Health Sciences, Sefako Makgotho Health Sciences University, Pretoria, South Africa; 3 Department of Health Sciences, Faculty of General Medicine, Keele University, London, United Kingdom

**Keywords:** addiction, dependency, nyaope, South Africa, qualitative study, risk factors, substance use, user perspectives

## Abstract

**Background:**

Nyaope increasing use has become a major public health concern, not only because of its detrimental health effects but also because of its far-reaching social consequences. This situation has caused distress for families, communities and the users themselves, contributing to ongoing cycles of dependency and instability at the social, family and individual levels. The aim of this study is to explore the perspectives of users regarding the factors underlying their use of and dependency on nyaope.

**Methods:**

An exploratory descriptive qualitative design was employed. Data were gathered from 10 nyaope users through semi-structured interviews.

**Results:**

Nyaope was widely and easily available, and purchases could be made with impunity. Factors such as peer pressure, boredom, poverty, unemployment and escapism all contributed to the initiation of nyaope use. Participants financed their habit through personal earnings, criminal activities and even support from family members. There was a pervasive sense of lawlessness associated with nyaope use, coupled with the stigmatisation and marginalisation of users by both their families and communities. Many users ultimately became part of a street-level nyaope brotherhood that perpetuated the use.

**Conclusion:**

Multisectoral and multifaceted interventions will be necessary to reduce the use of nyaope. Further research could quantify these factors and inform more effective prevention and treatment strategies.

**Contribution:**

These findings, along with input from community and family members, provide a comprehensive understanding of the factors related to nyaope use.

## Introduction

### Background

According to the World Health Organization,^1^ 38.4% of individuals with substance use disorders (SUD) use cannabis, 22.9% use methamphetamine, 18.8% use heroin and 5% cocaine. The South African National Drug Master Plan (DMP) 2006–2017 comprises a comprehensive and integrated set of strategies to address both the supply and demand issues.^2^ The above-listed substances, which go by various local names, such as dagga for cannabis, crack for cocaine and tik for methamphetamine, are primarily used in South Africa’s cities and large towns.^1^ In addition to these substances, there have been reports of a combination of two or more substances used in townships and other socio-economically disadvantaged communities. One such combination is nyaope, which is a novel psychoactive substance, used in South African cities and townships.^3^

Common in other South African cities, nyaope, which translates to ‘mixture’ in Tshwane Street slang, is also referred to as ‘brown sugar’.^4^ Nyaope is a relatively new and exclusive recreational street substance in South Africa. It first appeared in the Pretoria townships of Soshanguve and Mamelodi in the early 2000s, according to several media outlets.^4^ Despite having disastrous consequences in communities, nyaope took a long time to be criminalised by the authorities.^5^

Although it is believed that heroin is the primary ingredient in nyaope, the addition of antiretroviral medications has not been confirmed.^6,7^ The composition of nyaope is largely unknown. It is highly addictive, in addition to being readily accessible and reasonably priced in the townships, where even students in primary school can afford it.^2,4,6^ It has been claimed that the mixture, which is typically laced with low-grade heroin, can also contain milk powder, rat poison, bicarbonate of soda and pool cleaner.^3,4,6,7^ Nyaope is available for purchase as a dark powder that can be rolled with tobacco or marijuana and smoked, or it can be injected into a vein, often in an unsanitary and crude manner.^8^ Additionally, out of desperation and in disregard of all the consequences, some users inject themselves with the blood of another user who has recently taken nyaope, an action referred to as ‘*Bluetooth*’.^9,10^ It is easy to identify adolescents and young adults dependent on nyaope. They may be drowsy and dirty, congregated in Tshwane’s places of interest (such as train stations and taxi ranks) and begging for cash or doing piece jobs to buy their next fix.^4^

At individual, family and community levels, the use of and dependency on nyaope have extremely negative effects.^11,12,13,14,15^ Following experimental use, many users quickly become dependent. These individuals from mostly low-income backgrounds face financial difficulties; consequently, they begin by stealing from their homes or the neighbourhood and then discontinue their studies, lose their jobs and end up homeless.^10,11,12,13,14^

Nyaope users, who are mostly young and have dropped out of school, experience many complications.^4,10,11,12^ Because they find it difficult to service their dependency to nyaope, they resort to criminal activities to generate funds to buy the substance.^4,8,10,12^ Current evidence suggests that neither their family, the community or the police can find a sustainable solution.^10,11,12^ Users eventually develop new social connections in a ‘nyaope brotherhood’, which revolves around fellow users and individuals who facilitate access to nyaope.^4,8,9,10,12^ Numerous initiatives are emerging to support users because of the low recovery rate – less than 3% of nyaope users complete therapy and make a full recovery, and more than 40% discontinue the programme before it has been completed.^16,17^ It is crucial to pinpoint the main causes of dependency and provide more effective therapies. In this study, we aimed to explore the perspectives and experiences of nyaope users on the factors underlying the use of and dependency on the substance in Tshwane. Previous studies have explored the perspectives and experiences of community and family members.^10,12^

## Research methods and design

### Study design

We conducted a descriptive exploratory qualitative study using in-depth semi-structured interviews with nyaope users in Tshwane, South Africa.

### Setting

This study was conducted in Tshwane, home to a diverse population. Afrikaans, English, Sepedi, Sesotho, Tswana and Xitsonga are among the languages spoken there.^18^ Because of limited space in government rehabilitation centres, non-governmental and private rehabilitation facilities are becoming available.^19^ One of the non-government organisations that provide services to substance users and their families is the Community-Oriented Substance Use Programme (COSUP) which has 17 centres in Tshwane. Community-Oriented Substance Use Programme offers medical treatment, starts opioid substitution therapy (OST), follows up nyaope users and aims to find a solution to the nyaope issue.^17^ Community-Oriented Substance Use Programme started because of an agreement between the University of Pretoria and the City of Tshwane in 2016. Every centre has a social worker, a doctor, a clinical associate and one or more peer educators who are from the local community. Standard operating protocols have been designed for all staff levels to promote high quality care. These procedures include everything from client selection criteria to termination (either voluntarily or involuntary) and effective OST. Integrated medical, psychological and social screening is a part of identifying conditions and providing initial care.^16,17^

### Study population, sample size and approach to sampling

The study population included nyaope users with the following criteria: all nyaope users attending any of the 17 COSUP centres, aged 18 years and older, who had lived in Tshwane for at least 12 months and who had been using nyaope for at least 6 months. We excluded nyaope users who were high at the time of the interview or mentally impaired. Community-Oriented Substance Use Programme’s social workers assisted with the participant selection process. Users visited their COSUP site at least once a week, and over a period of 1-month, social workers identified and listed those who met the criteria. All eligible users were considered equally valid sources of information and were notified about the study, as well as the probable dates and times of interviews. Users indicated if they were willing to participate and then received follow-up phone calls or direct messages from their peers (recovered nyaope users) employed by COSUP. The final sample size of 10 participants was determined by data saturation, as there was no new information in the last two interviews.

### Data collection

The authors created an interview guide in accordance with the aim and objectives of the study as well as the risk and protective factors for substance use listed in the National DMP.^2,20^ The ecological theory of development that holds that a child meets many contexts throughout their lifetime that may influence their behaviour, such as family, school and communities,^21^ was also used to construct the interview guide. The opening question was: ‘Please elaborate on the risk and protective factors you have experienced in association with your use of nyaope’.

Individual, family, community and school factors were among the subjects that the guide covered. Participants were invited to their nearest COSUP sites for the semi-structured interviews. Interviews were conducted by a psychologist with training in qualitative interviewing. She spoke the common local languages fluently. The interviews were recorded and lasted between 60 min and 120 min.

### Data analysis

A qualified linguist translated the interviews from the vernacular languages into English and provided a verbatim transcript. The framework method was employed step by step,^22^ with the aid of NVivo 14.^23^ The first author familiarised himself with the qualitative data by reading the transcripts and observation notes and listening to the tapes. He created mutually exclusive codes and put them into organised categories using an inductive process to create a coding index. He then coded all the transcripts using the index and linking codes to manageable bites of text. He then created a series of charts that combined all data with the same code. Based on the coding index, he built charts corresponding to the major categories. Finally, he interpreted the data in each chart to identify themes as well as any relationships between themes. This entire process was supervised by Robert Mash and Toby Helliwell.

### Trustworthiness

The interviewer lived in Soshanguve, one of the townships affected by the nyaope phenomenon. Her training as a psychologist and in qualitative interviewing enabled her to isolate her own experiences and perspectives from the interviews. At the same time, her insider knowledge of the communities may have assisted an in-depth exploration of the phenomenon. The first author is a family physician who has previously been involved in phenomenological research^10^ and was working with COSUP. He had no clinical relationship with any of the participants. He paid attention to his own reactions, thoughts and feelings during the analysis process and worked under the supervision of the two other authors.

### Ethical considerations

Ethical clearance to conduct this study was obtained from the Stellenbosch University Health Research Ethics Committee (reference no.: 14848/S20/04/092 [PhD]). Additionally, permission was granted by COSUP’s management, and the study received approval from the Health Research Ethics Committee at Stellenbosch University.

## Results

Table 1 shows the characteristics of the 10 participants (eight men and two women). Seven themes are presented and summarised in Table 2.

**TABLE 1:** Characteristics of participants.

Participant	Gender	Age (years)	Townships	Years of nyaope use
1	Male	39	Soshanguve	15
2	Male	26	Soshanguve	10
3	Male	35	Mamelodi	9
4	Female	34	Mamelodi	10
5	Male	33	Soshanguve	17
6	Male	29	Eersterust	12
7	Male	39	Eersterust	9
8	Male	21	Winterveld	7
9	Female	32	Ga-Rankuwa	15
10	Male	29	Atteridgeville	13

**TABLE 2:** Themes.

Number	Theme
1	Easy access to a supply of nyaope
2	Reasons for using nyaope
3	The process of initiation to nyaope use
4	Funding for nyaope use
5	Nyaope use at school
6	No legal consequences for using and selling nyaope
7	Rejection of nyaope users

### Theme 1: Easy access to a supply of nyaope

Participants indicated that nyaope was widely available in their communities and that dealers could be reached without having to walk far. One participant explained:

‘Like, it is everywhere. They are selling it like they are selling sweets; you don’t even struggle to get it. You don’t even walk 5 to 10 minutes.’ (Participant 8, male, 21 years old)

Another participant indicated that the transaction between dealer and user was made openly:

‘You don’t even look back. You go straight to the dealer, and you buy … You don’t need to hide yourself … you go straight and buy.’ (Participant 4, female, 34 years old)

Another said that the price was affordable:

‘There is a packet for R25. Now they have reduced it; it is R18. Others can give it to you for R15. In Johannesburg, in Hillbrow, you can get it for R10.’ (Participant 1, male, 39 years old)

In addition to being accessible in public areas, nyaope was reported to be available in some unexpected locations, such as schools, prisons and the police station:

‘When I was in high school, there were children who were selling nyaope … they got them from outside people like their brothers, their uncles, their neighbours.’ (Participant 9, female, 32 years old)

‘Even in prison, I was smoking nyaope. Even police officers, they bring it to the prison. And the big ones … captains, they bring it. This thing (nyaope) is here, it is all over the place.’ (Participant 1, male, 39 years old)

‘There’s a time where I was in the police station here now, and I was [*having*] withdrawals. They were so severe … I thought I was dying. And the police officer asked me how much do I have? And I told him that I had about, I think, 120 rands. And he told me, “Give me the 100 bucks” … he only bought two (bags of nyaope); he gave me two, like, the police, he was in full uniform. I did not even feel like I was behind bars.’ (Participant 10, male, 29 years old)

### Theme 2: Reasons for using nyaope

Participants had a variety of motivations for using nyaope, many of which were intertwined with societal, economic and individual considerations. Several of the participants said that their social environment influenced them, such as friends or acquaintances who used nyaope. Peer pressure was thus a major factor in both starting and sustaining the use. For some, peer pressure was not an isolated factor, but was associated with other motivations. For example, one participant alluded to both peer pressure and unemployment:

‘I was influenced by a friend. I will say it was peer pressure … It made me sleepy and I felt good and I ended up saying this is a good thing to smoke … These things of nyaope … it is a lack of job opportunities. There are no jobs here at home.’ (Participant 6, male, 29 years old)

Another participant elaborated that they used nyaope because of peer pressure and boredom:

‘I chose the wrong path of listening to friends. I wanted to fit in and I felt comfortable when I was with friends. And when you want to fit in, you will want to do everything that they do and they like it … when I started smoking nyaope, I was in matric … I matriculated and after that I had nothing to do. I was always at home and not doing anything. That is when it escalated, and I started smoking a lot.’ (Participant 8, male, 21 years old)

Peer pressure and escaping life stress were also mentioned:

‘Peer pressure is one of them. I also wanted to fit in … Well, truly speaking, it was more of escaping the reality of my home, my family.’ (Participant 10, male, 39 years old)

The psychological effects of nyaope, such as feeling focussed, were also mentioned:

‘Because of concentration. Because when I wake up and I tell myself that I am fine, I would feel the heaviness on my eyes. But when you smoke nyaope, the feeling … it is good.’ (Participant 7, male, 39 years old)

Certain participants elaborated that their feelings of hopelessness and despair, stemming from their poverty and unemployment, prompted them to turn to nyaope:

‘… So, we started smoking nyaope because of my poverty. Poverty made me smoke nyaope. It’s the poverty … One plastic (one fix) can get us very high. We forget many things. We forget our problems there. It solved our problems every time.’ (Participant 3, male, 35 years old)

Some participants indicated that using nyaope was a coping mechanism:

‘My mother passed away from cancer … then my father passed away mysteriously … I can say this has affected me because I didn’t get help. There was no one to share with the pain I felt inside.’ (Participant 9, female, 32 years old)

One participant explained that she self-medicated with nyaope to sleep:

‘I told my friend that I have a problem of sleeping at night. And she said, “Oh, there is something that can make you sleep. Nyaope makes you sleep.” And I was not aware that one puff, then you are addicted for the rest of your life.’ (Participant 4, female, 34 years old)

The portrayal of substance use in the community and popular culture as glamorous and socially acceptable could normalise substance abuse:

‘I ended up thinking that smoking drugs is socially powerful. I ended up smoking too. When I was smoking, it was when I was able to spend time with the others who are smoking … I was engaging socially but with bad company … I thought the only way to be able to engage with people was to smoke drugs.’ (Participant 2, male, 26 years old)

### Theme 3: The process of initiation to nyaope use

There were different initiation trajectories. For some participants, initiation was direct, with nyaope being the first substance they ever used, while others initiated indirectly and experienced other substances before using nyaope:

‘It started from dagga … it went up to Mandrax, ecstasy. And after that, at the same time, it went to nyaope. I did a lot of drugs before nyaope. I tried so many.’ (Participant 5, male, 33 years old)

A surprising revelation was naïve initiation, in that some participants were not aware that they were using nyaope:

‘I don’t know if you are aware of it? It looks like Serokolo (Wild ginger or African ginger). So, I was telling myself that it was Serokolo only to find out that it was myaope … I didn’t know that it was nyaope.’ (Participant 1, male, 39 years old)

‘He told me it was dagga. I thought it was dagga, but it was mixed.’ (Participant 3, male, 35 years old)

### Theme 4: Funding nyaope use

Participants reported several funding mechanisms for buying nyaope. Many participants mentioned using more than one mechanism to maximise their funds. Broadly speaking, the funding mechanisms fell into two categories. The first category was non-criminal mechanisms, such as begging, pawning and selling one’s own goods:

‘You listen to them and be humble, but after you get the money, then you go. With what they are seeing, you pretend as if you are a good person. They will try to talk to you nicely because you know that you will get R50 or R30 … They bought me a phone and I sold it to get money to smoke nyaope.’ (Participant 1, male, 39 years old)

Participants misused pocket money for school or their college allowances. If they were still working, then they could self-fund their habit:

‘They (parents) would send me money for food, thinking that I was buying groceries, but I didn’t buy groceries, I was buying nyaope.’ (Participant 2, male, 26 years old)

‘Money, I was getting it from my parents, whereby they give me money to go and eat at school. Then I use it to smoke, not to buy food, lunch or something.’ (Participant 8, male, 21 years old)

Some participants even received financial support from their parents to sustain their nyaope use to protect them from criminal activities and community justice:

‘My mom used to give me money to buy nyaope, so that I don’t steal.’ (Participant 6, male, 29 years old)

‘Yes, it was bad for us. All of us. They [*parents*] protected me because the community wanted to hurt all of us. So, we had to move. So, they [*parents*] would give me money for nyaope.’ (Participant 3, male, 35 years old)

The second category of funding mechanisms is related to criminal activities. This usually involved stealing from home to resell. One participant indicated keeping a permanent inventory of potential goods in their home that could be sold without suspicion or detection:

‘At home there was other stuff which they were not using … I sold them and got money to go and buy nyaope.’ (Participant 1, male, 39 years old)

Another participant reported scanning people in the community for any opportunity to steal:

‘While people are drinking alcohol and enjoying themselves, you are looking at their bags, their phones, everything, you take.’ (Participant 6, male, 29 years old)

Participants also reported mugging and breaking into people’s homes:

‘When I need money to smoke or to support my habit, I must go and rob some people or break into someone’s yard or do something bad to provide for my habit.’ (Participant 3, male, 35 years old)

‘We broke into offices in Garsfontein and we took the laptops. After we took them, then we would go and sell them … to satisfy the habit.’ (Participant 1, male, 39 years old)

It was also reported that shop owners sometimes requested the theft of specific items:

‘And others, they can also tell you that they want this and that and you can find it at this place, then you will go and steal it. Then I got money from the shop.’ (Participant 7, male, 39 years old)

We noted that most of the transactions to trade stolen items were not financially advantageous for the nyaope users. However, because of their need for money to purchase their fix, they had to take any offer. In this regard, participants stated:

‘But others they steal. So, there are people who know that people who are smoking, even if they have an expensive item, they will not sell at a high price, because all they want is nyaope. I am sorry to say this. There is this one guy we found, and he was drunk and lost. We went into his car trying to give him direction, but instead we ended up chasing him out of his car … We took his car and we sold it for only R3000 … And what is sad is the treatment from the sellers. There is no credit, and you can spend R2000 today and tomorrow when you come back and you don’t have money, they won’t give you.’ (Participant 7, male, 39 years old)

‘As I have also said earlier, people use people who are smoking. If you have a phone [*for sale*], you will ask for R200, but they can end up paying you R50.’ (Participant 10, male, 29 years old)

### Theme 5: Nyaope use in schools

Participants reported many issues regarding their time at school. This included the availability of nyaope at school and school premises being used as an initiation platform:

‘I don’t know if I am correct. But I will say the school contributed … We even used to smoke in the toilets in school and others learned how to smoke in the school … And you find that the seller is an adult, but they will use a school child to sell in the school …’ (Participant 7, male, 39 years old)

Truancy within school was also a problem and the school premises were used as a place to hide and enjoy nyaope:

‘They said that most of the time I was absent from school, I was not attending well and things were just messed up. Yes, I was going to school every day, but I was not going to the class every day. I will end up in the school yard or the toilets. That is where we were hiding.’ (Participant 6, male, 29 years old)

The use of nyaope led to dropping out of school for various reasons. One participant indicated not being able to cope because of withdrawal symptoms:

‘How could I concentrate at school with stomach cramps? Yes, it was grade 10 … When I left school in grade 10, I felt like my things are going well. I was no longer going to school; I only focused on feeding my craving …’ (Participant 1, male, 39 years old)

The inability to cope with school discipline caused some to drop out:

‘I was in grade 10 … All I was thinking was to get high. I even dropped out of school because the other teacher was always after my case … Like, maybe, when I am late at school, that teacher will follow me asking why I am late, why didn’t I do my homework … I said, eish, this teacher is after me. So, I dropped out of school. I was not interested anymore …’ (Participant 4, male, 34 years old)

The expulsion was another consequence:

‘Because the nyaope thing was controlling me … Like, in the morning, if I didn’t smoke, I can’t attend classes. I have to make sure that I get money and go to smoke first and come back to school. But when I come back, it is already late. That time, maybe, it is school out, we are going home. Then, which means that day I didn’t attend classes, I ended up in grade 10 … At school, I started stealing other children’s cell phones. They ended up expelling me, telling me that you are no longer accepted here at school.’ (Participant 8, male, 21 years old)

### Theme 6: No legal consequences for using and selling nyaope

Some participants had the perception that dealers were working with police officers:

‘When it comes to drugs, they (police) don’t help because … there is a place I used to go at the hostel; the police would come there and us who are buying small quantities; when they see you, they can even beat you up. But when they get to the seller, the mood changes. Everything is fine, they are laughing … Even if they can send the police out to patrol, the sellers will get a tip-off.’ (Participant 7, male, 39 years old)

‘Truly speaking, the police are also working with those people who are selling nyaope. Because, instead of arresting them, they just come and take a bribe and go.’ (Participant 8, male, 21 years old)

Participants reported that police did not see the point in pursuing cases related to the use of nyaope, and that neither the dealer nor the user was arrested or prosecuted:

‘[*The*] nyaope thing now is so out of control that if the cops see you using nyaope, they don’t arrest you anymore. They just take you in, beat you and let you go … In X magistrate court, if they arrest you with nyaope, tomorrow morning, you are out. Not even on bail or anything, we just walk out, that is called the white door … You don’t even see the magistrate, you just walk out straight … You don’t even see the magistrate, they call it a waste-of-time crime.’ (Participant 10, male, 29 years old)

A considerable number of the participants had served time in prison for secondary offences, such as theft and burglary. None of them reported ever being questioned or prosecuted by police regarding the use and possession of nyaope:

‘The only time cops arrest you and prosecute is for theft and going upwards … not for nyaope … I went to prison in 2006 and came back in 2015. They had sentenced me to 13 years for theft.’ (Participant 1, male, 39 years old)

‘After I relapsed in 2010, I was not very fortunate. We were hustling and stealing. Anything we come across; it was cars, shop burglary … we could steal and have a lot of money … but then one day, I got arrested for a car theft.’ (Participant 2, male, 26 years old)

### Theme 7: Rejection of nyaope users

Participants felt rejected and shunned by their communities because of their nyaope use:

‘They treat us badly, badly. They don’t think we are human beings. We are just like criminals; we are just like nobody. Just like a stranger outside the community. The community doesn’t like nyaope at all, doesn’t like addicts at all. They treat us badly, badly.’ (Participant 3, male, 35 years old)

Some residents found nyaope users undesirable and saw them as potential thieves:

‘The treatment is horrible … they don’t want to hear anything from somebody who is using. They treat them bad … they can beat you and kill you. They don’t even mind. Even though maybe you, like, want a piece job from your neighbour, he or she won’t trust you because you are an addict; you are smoking nyaope. He will think that you are going to steal from him.’ (Participant 4, male, 34 years old)

Some participants felt that they were being taken advantage of because they were willing to work hard to acquire cash to satisfy their cravings. Community members would offer them small amounts of money to work as cheap labour:

‘In the community, they used to call me more often to come and help them at their homes and they will give me small amount of money. I had to work for a lot of different people for me to put money together for that day to have a smoke. I would ask for odd jobs and work. Then when I am done and they pay me, I will go and buy nyaope.’ (Participant 6, male, 29 years old)

‘In the community, some people like to use people who are smoking nyaope. Like, we don’t hustle the same way. Others are doing gardens, then they use you in that way … And others, they are happy, because they will use you to do their gardens, while their children are sitting there and relaxing, and after that they will give you R30 (the price of one bag of nyaope) because you are smoking. That is why I said others are using us. Instead of them feeling for you as a parent, no, they see an opportunity for using you, while their children are not doing anything.’ (Participant 7, male, 39 years old)

A second form of rejection was by participants’ families. Participants mentioned experiencing physical or emotional harm from family members:

‘The family chased me away from the house; I used to sleep on the streets or in jail.’ (Participant 1, male, 39 years old)

‘Imagine they came looking for me and said, “We are going to beat him.” … I was begging my relatives not to allow them to take me out my home. And they said that, “Maybe you will learn,” and I said, “What if I die?” And they said, “If that’s the price you must pay, then let it be.” So, and I asked myself is my life that worthless … they believe more in the mob justice because I embarrassed them so much … their first child to ever smoking. Instead of looking at the root of the problem, they threw me to the wolves and said that if he lives, imagine if he dies, so be it.’ (Participant 10, male, 29 years old)

Another participant’s mother welcomed the court decision for him to be in prison, which left him feeling abandoned:

‘After I got arrested, my mom said she is tired; she wants me to spend some time in prison.’ (Participant 2, male, 26 years old)

Such rejection could push users into developing stronger bonds with other nyaope users:

‘I was feeling great, happy, with nyaope guys. I was feeling at home, I was welcomed. I was feeling the comfort. I felt relief when I was with those guys of nyaope.’ (Participant 4, male, 34 years old)

## Discussion

### Discussion of findings

Easy access, affordability, peer pressure, normalisation, boredom, poverty, self-medication and coping with stress were all mentioned as reasons to start using nyaope. Buying and selling took place publicly and with impunity, even in schools. In these poor communities, the habit was maintained by piecework, selling possessions and engaging in criminal activity. Only thefts and burglaries appeared to result in legal action, while the trade in and use of nyaope did not. There were concerns about police attitudes and even corruption in this regard. Dependency and its consequences eventually led to rejection by families and community members and users lived on the streets, supported by other users in a nyaope brotherhood. These factors were involved in sustaining this cycle from the initiation to becoming part of the nyaope brotherhood.

Nyaope use has serious clinical repercussions, especially because of the high-risk behaviours involved with its use.^4,10,12^ Many users inject the substance, frequently sharing needles or injecting with non-sterile methods, which greatly raises the risk of human immunodeficiency virus (HIV) and bloodborne illnesses such as hepatitis B, C and D. The immunosuppressive effects of prolonged drug use put users at additional risk for opportunistic infections, including pneumonia, TB and other respiratory tract infections and infective endocarditis, a potentially lethal illness brought on by bacterial infections that damage the heart valves.^4,8^ Delays in diagnosis and poor treatment outcomes result from these health problems, which are made worse by inadequate cleanliness, hunger and limited access to healthcare services.

Our findings suggest that nyaope is widely available in diverse locations and that users may readily acquire this harmful substance, which is consistent with current nyaope literature.^4,8^ Findings also shed light on the public nature of transactions between dealers and customers, as well as the affordability of the substance. Most notably, our study revealed that nyaope is not only ubiquitous in public venues but has also found its way into schools, as previously documented,^9,10,13^ and raises concerns about young people’s susceptibility to substance dependence. Furthermore, reports of nyaope being available in police stations and prisons highlight the challenge of maintaining control over the supply of nyaope. The comparatively low cost of the substance makes it more available to a wider range of people,^4^ which adds to the normalisation of substance use within communities, rather than seeing nyaope as a serious threat to public health. There is a critical need for a comprehensive and coordinated national policy or framework aimed at addressing the growing crisis of nyaope use in South Africa. The current response remains fragmented and reactive, often limited to short-term interventions that fail to address the root causes and long-term consequences of addiction. A robust framework should integrate prevention, early intervention, cessation, harm reduction and rehabilitation, tailored specifically to the unique context of nyaope users, many of whom are young people living in underserved communities.

Prevention efforts must be community-based and youth-focussed, raising awareness about the dangers of nyaope and building protective social structures. For those already using the substance, the policy should ensure equitable access to medically supervised detoxification, psychosocial support and evidence-based treatment programmes. Rehabilitation should go beyond clinical care to include skills development, educational support, employment opportunities and family reintegration, thereby addressing the social determinants that drive relapse. Importantly, the policy must promote multi-sectoral collaboration, bringing together the departments of Health, Social Development, Education, Justice and local government, as well as NGOs and civil society organisations. By developing and implementing a nationally endorsed, well-resourced and culturally sensitive framework, South Africa can begin to reduce the burden of nyaope use and restore hope to affected individuals, families and communities.

Although participant reports indicate that there are wide variations in the reasons for starting to use nyaope, a noteworthy finding in this study is the emerging use of nyaope as a form of self-medication for headaches and insomnia, according to recent literature on nyaope.^4^ This novel aspect adds to the complexity of understanding initiation behaviours, suggesting that beyond recreational or social motivations, some users perceive nyaope as a remedy for certain physical ailments. This points to the need for targeted interventions to communities, addressing both social determinants and misinformation about the commencement of nyaope use. Furthermore, the vertical and horizontal initiation described by nyaope users is consistent with previous literature, which indicates that social and environmental factors play an important role.^24^ Acquaintance with other users and living in places with easy access to nyaope were commonly mentioned as important factors.^4,8,9,10^ This finding is consistent with previous research that has highlighted the role of peer pressure and the availability of substance use initiation.^24^

Participants indicated that the most important way of acquiring cash was the resale of stolen commodities. This is consistent with previous research indicating that nyaope users engage in illegal activities to fund their substance use and dependency.^11,13^ We also found that participants felt exploited by some community members and shop owners. In rare cases, business owners may even prompt theft of a specific item. This would imply that the community is also contributing to the problem in two ways. Firstly, by underpaying nyaope users for work done and, secondly, by purchasing second-hand products, knowing that they are most likely stolen items.

Furthermore, there appears to be a perception of collaboration between certain police officers and nyaope dealers, which is corroborated by other studies.^10,11^ Communities impacted by nyaope use live in fear of reprisals from nyaope dealers, are becoming more frustrated and have less faith in law enforcement.^10,12^ Although it is important to acknowledge that perceptions may not always correspond with reality, the apparent lack of arrests and prosecutions of both users and dealers emphasises the necessity of conducting a comprehensive inquiry into attitudes and possible corruption within law enforcement institutions. Although the nyaope phenomenon has existed in Tshwane for more than two decades, nyaope use and trade were criminalised only much later, after they had already resulted in devastating individual, familial and societal effects.^5^ Still, nyaope sellers and consumers appear to operate with impunity. Most participants stated that they had done time in prison for prior offences that had nothing to do with using nyaope specifically, but with obtaining money for nyaope purchases. None of the participants indicated having been asked direct questions by the police concerning their use and possession of nyaope. This finding presents a serious obstacle to reducing nyaope use and trade. It poses questions about the tactics and priorities used by law enforcement to combat the nyaope epidemic. Improving public confidence in law enforcement and reducing the negative impacts of nyaope on communities depend on tackling these issues. It will require more research, more community involvement and more focussed law enforcement actions.

The last component of the framework is the constitution of the nyaope brotherhood. This community-based network is composed of nyaope users sleeping on the streets and having trouble coping with dependency issues. The prevalence of familial rejection among users is high.^11,13^ Familial rejection can be one of the main factors in pushing the users to the street, followed by community rejection.^10^ Because stealing from family members to support their habit results in conflicts and dysfunctional families, users frequently find themselves alone and alienated from their families. The void left by this rejection, whether genuine or perceived, could be filled by the brotherhood’s members’ giving alternative emotional support, empathy and companionship. This fosters a sense of solidarity among persons who are marginalised by society at large. An environment is created in which it is easier to purchase nyaope by combining resources and raising money in the street. This cooperation keeps users in the brotherhood in a loop of mutual reliance, while also guaranteeing a consistent supply of the substance. Without considering the knowledge of how the brotherhood came to be, as well as its inside dynamics, it would be challenging to create strategies to counteract the use of nyaope.^16,17^

**FIGURE 1:**
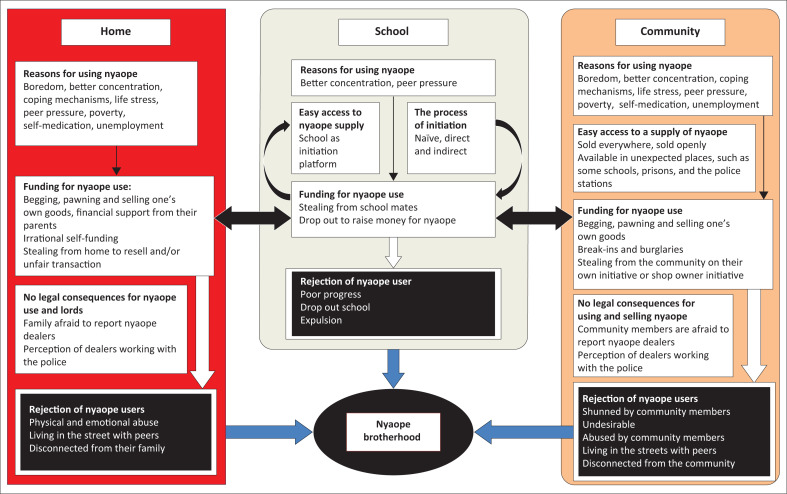
An emerging conceptual framework of factors contributing to the use of nyaope.

The current state of nyaope use in Tshwane can be attributed to a confluence of factors that drive both the availability and consumption of the substance. The network of nyaope suppliers, including its availability in unexpected locations such as schools and police stations, was made clear by this study. Participants’ socio-economic and personal motivations for using nyaope included peer pressure, poverty, unemployment and boredom. Initiation could be naïve, direct or indirect via other substances. Nyaope users funded their habit from their own income as well as illegal activities or other sources. We also found that the participants experienced stigmatisation and marginalisation from their families and communities, and that there was a sense of impunity regarding the police and law connected to nyaope use. The ways in which these and previously described factors^10^ are connected are presented in Figure 1.

Despite growing concern around the widespread use of nyaope, there remains a significant gap in research that limits the development of effective prevention and treatment strategies. Much of the existing literature is either anecdotal or focussed on general substance use, leaving nyaope-specific issues poorly understood. There is a need for in-depth studies that explore the socio-economic drivers behind nyaope use, such as poverty, unemployment and community disintegration. Additionally, research should investigate the long-term physical and mental health consequences, the effectiveness of current rehabilitation approaches and the reasons for high relapse rates. Another key area needing exploration is the pattern of use among different demographic groups, including adolescents, women and people living with HIV. There are also unanswered questions about how nyaope users navigate healthcare systems, what barriers exist to accessing treatment and what community-based models might better support recovery and reintegration. Addressing these gaps through interdisciplinary and context-sensitive research will provide critical evidence to inform policy, improve service delivery and ultimately guide more targeted and impactful responses to the nyaope crisis.

### Strengths and limitations

All the participants were receiving services at COSUP centres and were therefore in recovery or rehabilitation. While this may have enabled reflection on their narratives and participation in the study, we did not obtain data from users who were not connected to treatment and who may have had different perspectives. Also, most participants were male, as most users are male; women might have had a different experience.

### Implications

The findings of this study have the following implications:

- A favourable relationship between law enforcement and the public must be fostered and community policing efforts enhanced. To address issues and build trust, frequent community gatherings, public forums and cooperation between the local police and community members must be encouraged. Additionally, there is a need to handle any potential corruption or collusion between police and nyaope users and sellers. Focussed community outreach by police members and community leaders can increase awareness about refraining from purchasing stolen goods.
- Rehabilitation initiatives should take cognisance of the factors related to nyaope use and address these in counselling and treatment plans (peer pressure, rejection from the family and the community, funding for nyaope use). These initiatives must work together with community organisations and medical specialists to offer comprehensive help to people attempting to overcome dependency. A strong intersectoral approach between health and social services as well as police and education is clearly needed.
- Importantly, the formation of nyaope brotherhoods should be prevented by trying to retain relationships with families and social networks. Community-driven programmes should be established to help families with members who are nyaope users. These programmes must encourage families to avoid rejecting these members and inform the local community about the opportunities to address the underlying socio-economic factors that lead to the marginalisation of nyaope users. One should discuss the broader social determinants and how these are important. These determinants include poverty, boredom and poor educational opportunities. In a society with 40% unemployment among youth and a sense of futility for one’s future, it is not surprising that crime, gangsterism and drug use take hold. There is a need to improve the social foundation of society with educational opportunities and employment.
- Further studies can be conducted to quantify the qualitative findings from this and the previous studies in this research project (Nzaumvila et al. 2023).

## Conclusion

Based on the findings, it is clear that nyaope use is deeply embedded in the socio-economic realities of the communities where it is most prevalent. Easy access, low cost and the open, unchecked trade of the substance – even in schools – highlight the failure of current law enforcement and public health systems to adequately respond to this crisis. In contexts of poverty, unemployment and social exclusion, nyaope becomes more than a drug – it becomes a coping mechanism for stress, boredom and hopelessness. The normalisation of use, particularly among youth, is further reinforced by peer pressure and the absence of meaningful alternatives, such as education, job opportunities or recreational activities.

The fact that criminal activities such as theft and burglary are punished, while substance dealing and use are largely overlooked, reveals significant gaps in policy implementation and justice, further undermining trust in public institutions. The ongoing cycle of selling possessions, doing piecework or committing crimes to sustain the habit highlights the urgent need for a comprehensive, multi-sectoral response that addresses not only substance use and dependency but also the underlying socio-economic drivers. Any long-term solution must integrate poverty alleviation, community empowerment, education and accessible rehabilitation services, if we are to disrupt the cycle of dependency and restore hope in these marginalised communities.
